# Temporal changes in fecal microbiota of patients infected with COVID-19: a longitudinal cohort

**DOI:** 10.1186/s12879-023-08511-6

**Published:** 2023-08-18

**Authors:** Tatiana Galperine, Yangji Choi, Jean-Luc Pagani, Antonios Kritikos, Matthaios Papadimitriou-Olivgeris, Marie Méan, Valentin Scherz, Onya Opota, Gilbert Greub, Benoit Guery, Claire Bertelli, the RegCOVID Study Group, Pierre-Yves Bochud, Florian Desgranges, Paraskevas Filippidis, David Haefliger, Eleftheria-Evdokia Kampouri, Oriol Manuel, Aline Munting, Jean Regina, Laurence Rochat-Stettler, Veronique Suttels, Eliana Tadini, Jonathan Tschopp, Mathias Van Singer, Benjamin Viala, Peter Vollenweider

**Affiliations:** 1 Service of Infectious Diseases, Lausanne University Hospital and University of Lausanne, Rue du Bugnon 46, BH10-553, 1011 Lausanne, Switzerland; 2 Institute of Microbiology, Lausanne University Hospital and University of Lausanne, Lausanne, Switzerland; 3 Service of Intensive Care, Lausanne University Hospital and University of Lausanne, Lausanne, Switzerland; 4 Division of Internal Medicine, Lausanne University Hospital and University of Lausanne, Lausanne, Switzerland

**Keywords:** COVID-19, SARS-CoV-2, Gut microbiota, Microbiota profiling, Gut-lung axis

## Abstract

**Background:**

Severe acute respiratory syndrome coronavirus 2 (SARS-CoV-2) is a multifaceted disease potentially responsible for various clinical manifestations including gastro-intestinal symptoms. Several evidences suggest that the intestine is a critical site of immune cell development, gut microbiota could therefore play a key role in lung immune response. We designed a monocentric longitudinal observational study to describe the gut microbiota profile in COVID-19 patients and compare it to a pre-existing cohort of ventilated non-COVID-19 patients.

**Methods:**

From March to December 2020, we included patients admitted for COVID-19 in medicine (43 not ventilated) or intensive care unit (ICU) (14 ventilated) with a positive SARS-CoV-2 RT-PCR assay in a respiratory tract sample. 16S metagenomics was performed on rectal swabs from these 57 COVID-19 patients, 35 with one and 22 with multiple stool collections. Nineteen non-COVID-19 ICU controls were also enrolled, among which 14 developed ventilator-associated pneumonia (pneumonia group) and five remained without infection (control group). SARS-CoV-2 viral loads in fecal samples were measured by qPCR.

**Results:**

Although similar at inclusion, Shannon alpha diversity appeared significantly lower in COVID-19 and pneumonia groups than in the control group at day 7. Furthermore, the microbiota composition became distinct between COVID-19 and non-COVID-19 groups. The fecal microbiota of COVID-19 patients was characterized by increased *Bacteroides* and the pneumonia group by *Prevotella*. In a distance-based redundancy analysis, only COVID-19 presented significant effects on the microbiota composition. Moreover, patients in ICU harbored increased *Campylobacter* and decreased butyrate-producing bacteria, such as *Lachnospiraceae*, *Roseburia* and *Faecalibacterium* as compared to patients in medicine. Both the stay in ICU and patient were significant factors affecting the microbiota composition. SARS-CoV-2 viral loads were higher in ICU than in non-ICU patients.

**Conclusions:**

Overall, we identified distinct characteristics of the gut microbiota in COVID-19 patients compared to control groups. COVID-19 patients were primarily characterized by increased *Bacteroides* and decreased *Prevotella*. Moreover, disease severity showed a negative correlation with butyrate-producing bacteria. These features could offer valuable insights into potential targets for modulating the host response through the microbiota and contribute to a better understanding of the disease's pathophysiology.

**Trial registration:**

CER-VD 2020–00755 (05.05.2020) & 2017–01820 (08.06.2018).

**Supplementary Information:**

The online version contains supplementary material available at 10.1186/s12879-023-08511-6.

## Background

The severe acute respiratory syndrome coronavirus 2 (SARS-CoV-2) emerged in Wuhan in early December 2019 [1] and was rapidly identified as a novel betacoronavirus [2]. Initially considered as a respiratory disease, it rapidly became clear that coronavirus disease (COVID-19) was a systemic disease potentially involving all organs, including the gastrointestinal tract [3].

Although the primary location of the SARS-CoV-2 infection is clearly the respiratory tract, an array of observations stages the gastrointestinal tract as an important factor to disease development and immune response [4–7]. Clinically, the significant prevalence (17.6%) of digestive symptoms was reported in a meta-analysis regrouping the results of 60 studies and 4243 patients [8]. The involvement of the gastrointestinal tract was displayed in the same study by the high positivity (48.1%) of stool samples, even when respiratory samples became negative [8]. At the molecular level, gut involvement can be explained by a high-level expression of angiotensin-converting enzyme 2 (ACE2) in the small intestine, kidney, gallbladder, testis, and colon onto which SARS-CoV-2 spike proteins bind prior to cellular fusion [9]. These observations suggest the likely role of the gut microbiota in the course of severe forms of COVID-19 [6, 10, 11].

The human gut microbiota, comprising bacteria, archaea, fungi and viruses, has been extensively described [12]. It is mainly composed of strict anaerobes which dominate facultative anaerobes and aerobes [13]. Among more than 50 bacterial phyla, only three dominate: *Firmicutes*, *Actinobacteria* and *Bacteroidetes* represent over 80% of the microbial population. *Proteobacteria* and *Fusobacteria* are found in small proportions. Several studies showed that the microbiota can influence host health and change the course of diseases [14]. The intestine is a critical site of immune cell development controlling both intestinal and extra-intestinal immunity. Several evidence suggest that the gut microbiota plays a key role in the adaptation of the lung immune response [15, 16]. Gut microbiota could hence play a major role in SARS-CoV-2 infection where the host immune response prevails [17].

To our knowledge, a limited number of studies have evaluated the dynamic of the gut microbiota in patients infected with SARS-CoV-2 [18–22]. A cross-sectional study compared COVID-19 to H1N1 patients and healthy controls, highlighting a decrease in diversity and an increase in opportunistic pathogens in COVID-19 compared to healthy controls [18]. An increase of bacteria such as *Clostridium hathewayi*, *Actinomyces viscosus*, or *Bacteroides nordii* [20, 23–25] and a decrease of butyrate-producing bacteria such as *Fecalibacterium prausnitzii, Clostridium butyricum, Clostridium leptum,* and *Eubacterium rectale* [6, 18, 20] was underlined in several studies. Overall, compared to healthy controls, the microbiota of COVID-19 patients was characterized by a depletion of beneficial commensals and an enrichment of opportunistic pathogens.

This longitudinal observational study aimed at describing the fecal microbiota profile in COVID-19 patients admitted to intensive care unit (ICU), or in medicine wards, and evaluating potential correlations between fecal shedding of the virus and disease severity. We also compared the gut microbiota of COVID patients with critically ill patients admitted in the intensive care unit due to bacterial pneumonia or non-infectious diseases requiring ventilation as control groups.

## Methods

### Study setting

This study took place in Lausanne University Hospital (CHUV), a one-thousand-bed tertiary university hospital in Lausanne, Switzerland.

### Study design and participants

This prospective observational study included all adult patients consecutively hospitalized with a confirmed SARS-CoV-2 infection from March through December 2020.

Patients fulfilling the following criteria were included: age > 18 years-old, symptoms associated with Covid-19, and admitted for this infection in medicine or in ICU, positive reverse-transcriptase-polymerase-chain-reaction (RT-PCR) assay for SARS-CoV-2 in a respiratory tract sample, and a written informed consent. Exclusion criteria were: pregnant or lactating women, known with Inflammatory bowel disease or irritable bowel syndrome, no consent of the patients or inability to provide consent from a next of kin.

Critically ill non-COVID patients with or without pneumonia were included as controls. Patients were recruited in ICU provided they were antibiotic-naïve for three months upon admission, intubated for less than 48 h and with an anticipated length of stay of more than 48 h. Informed consent was obtained from patients’ next-of-kin and confirmed by patients whenever possible. Exclusion criteria were: age < 18 years old, antibiotic treatment in the previous 30 days, immunosuppression (patients with solid organ or stem cell transplantation, HIV-positive patients with detectable viral load, prednisone > 0.5 mg/kg, immunomodulatory treatment or recent chemotherapy) or inclusion in other interventional studies. Patients who died during the first 24 h from inclusion were also excluded. Patients were classified based on the development or absence of infection during their ICU stay as follows: 1) patients with ventilator-associated pneumonia (VAP) treated with antibiotics (pneumonia group), 2) patients without infection (control group). VAP was defined as a clinical suspicion of pneumonia developing ≥ 48 h after endotracheal intubation and presence of new or progressive pulmonary infiltrates on chest radiograph at least one the following: 1) fever, 2) peripheral leukocytosis, 3) purulent tracheal secretions or 4) decline in oxygenation [26].

### Inclusion and data collection

All SARS-CoV-2 infected patients admitted at CHUV were assessed for recruitment in this observational study through the project leader or study nurses. The list of SARS-CoV-2 PCR positive patients was updated every day from the laboratory and was available onto the electronic health record (EHR) software for review by infectious disease physicians. The consent was obtained by a dedicated team of infectious diseases clinicians. Informed consent was obtained from all subjects and/or their legal guardian(s).

Stool samples were collected with sterile single-use rectal swab (Liquid Amies Elution Swab Collection and Transport System, eSwab 490CE, COPAN) at the diagnosis of SARS-CoV-2 and 7, 14, 21 and 28 days of SARS-CoV-2 diagnosis (+ 2 days). A swab moistened with liquid amies medium was inserted 1–2 cm past the anal verge and was gently rotated 360.

The CHUV EHR provided epidemiological, clinical, radiological and laboratory data. Epidemiological data included age, sex, height, weight, and relevant comorbidities including a Charlson Comorbidities Index (CCI). We collected data on clinical presentation, SARS-CoV-2 treatments, antibiotics administration at sampling and within 30 days before, other concomitant therapies, non-pharmacological interventions and clinical course within CHUV. To assess severity, we calculated quick Sequential Organ Failure Assessment (qSOFA) score, Confusion/Respiratory rate/Blood pressure/age ≥ 65 years (CRB-65) score and National Early Warning Score (NEWS) [27–29].

Laboratory data included full blood count, D-dimers, creatinine, highly sensitive cardiac T-troponin, C-reactive protein (CRP), procalcitonin (PCT), ferritin, liver function tests, blood type and real-time PCR to detect SARS-CoV-2 [30] in respiratory and stool samples.

For ICU controls, rectal swabs were collected within 48 h of intubation with subsequent sampling performed weekly thereafter provided the patient was still hospitalized. Specimen collection was repeated on the day of antibiotic introduction for clinical suspicion of infection and subsequently 5 days later. Finally, specimens were collected upon extubation and ICU discharge. Rectal swabs were (DNA/RNA Shield Collection Tube w/Swab, Zymo Reearch, CA, USA) were inserted into the anal canal, beyond the anal verge (± 3 cm). Swabs were rotated gently and then removed.

For all patients, clinical, laboratory and radiological data were collected from the electronic health record and entered in an electronic clinical report form (eCRF) using the REDCap platform (Research Electronic Data Capture v8.5.24, Vanderbilt University, Tennessee, USA) [31].

### Statistics on clinical and demographic data

Statistical analyses were performed using R software v3.6.2 (R Foundation for Statistical Computing). Categorical variables were presented as percentages (numbers), normally distributed continuous variables were presented as mean ± standard deviation (SD) and continuous variables with a skewed distribution were presented as median [interquartile range (IQR)].

For the descriptive analysis, proportions of categorical variables were analyzed using chi-square goodness of fit test; we used Student t-test for normally distributed variables or Mann–Whitney-Wilcoxon test for continuous variables with a skewed distribution. For inflammatory biomarkers, continuous variables were converted into categorical variables using cut-off values from the literature [32]. We did not impute any values for missing data.

### DNA extraction and SARS-CoV-2 PCR

The diagnosis of SARS-CoV-2 was made using three different diagnostic methods of comparable performance, including our in-house TaqMan molecular diagnostic platform, the GeneXpert plateform (Cepheid, Ca, USA) and the cobas 6800 and cobas Liat (Roche, Basel, CH) [33–35]. For the in-house molecular diagnostic platform, DNA was extracted from rectal swabs collected in DNA/RNA Shield Collection Tube (Puritan, Guilford, USA) on a MagNA Pure automated platform (Roche, Basel, Switzerland). SARS-CoV-2 RT-PCR was performed on the in-house automated molecular diagnostic platform targeting the RdRp and the E genes [30, 36, 37]. For the E gene, we used the primers targeting the E gene using the primers and probes described by Corman and colleagues [30] and the amplification program described by Opota et al. [33]. For the PCR targeting the RdRP gene, the primers were adapted from Corman et al., Muenchhoff et al. and Pillonel et al. as follow: forward primers RdRP_SARSr-Fmod 5’- AAATGGTCATGTGTGGCGGT-3’, reverse primers RdRP_SARSr-Rmod 5’-GTTAAAAACACTATTAGCATAAGCAGTTGT-3’ and two probes RdRP_SARSr-P2 5’-FAM- CAGGTGGAACCTCATCAGGAGATGC-BHQ-1–3’ and RdRP_SARSr-P3 5’-FAM- CCAGGTGGWACMTCATCMGGWGATGC—QSY-3’ [30, 35, 36]. The cycle threshold of the E gene RT-PCR was converted into viral load using the formula logVL = -0.27Ct + 13.04, as previously reported [33].

### 16S rRNA metagenomic sequencing

Amplification of the V3V4 region of the 16S rRNA gene and library preparation were performed according to the protocol ‘16S Metagenomic Sequencing Library Preparation’ (Part. #15,044,223 Rev. B, Illumina, San Diego CA, USA). Briefly, the V3V4 region of the bacterial 16S rRNA gene was amplified through 25 PCR cycles. Sample-specific barcode and adapter sequences were attached to the amplicons by 8 extra PCR cycles. The quality of PCR products was evaluated measuring their length and abundance using a Fragment Analyzer with the Standard Sensitivity NGS kit (Agilent, Santa Clara CA, USA) and their concentration on a Qubit with the dsDNA High Sensitivity kit (Thermo Fisher Scientific, Waltham MA, USA). Batches of libraries were prepared on a Microlab Starlet robot (Hamilton, Bonaduz, Switzerland). Each run included one positive extraction control consisting of the MSA-2002™ mock community (ATCC, Manassas, USA) in order to assess the efficiency of the whole process from extraction to sequencing. One extraction negative control and one library negative control were also included to track contaminations. Libraries were normalized to the same concentration, pooled together, and sequenced on an Illumina MiSeq (Illumina, San Diego, USA) with V3 Reagent kit to generate 300 bp paired-end reads.

### Microbiota analysis and visualization

Raw sequences in FASTQ files were processed using an in-house bioinformatics pipeline, named zAMP for routine 16S microbiome analysis (https://github.com/metagenlab/zAMP release v 0.9.15), implementing DADA2 (v 1.12.1) [38] algorithm in a Snakemake [39] pipeline, before further analysis and visualization in R (v. 4.1.1) using vegan and ggplot2 packages. Briefly, once PCR primer sequences were trimmed with Cutadapt (v 2.10) [40], DADA2 filtered out reads predicted to have over 6 sequencing errors based on their Phred score, corrected errors using the parametric error model for each sequencing run, merged paired reads, generated amplicon sequence variants (ASVs), filtered sequences by length (between 390 and 480 bp), and removed chimeras. These error-corrected ASVs were classified by the RDP classifier [40] in QIIME (v 1.9.1) [41] with the EzBioCloud reference database (2018.05 release, pre-processed to integrate the taxonomic identification resolution of V3V4 sequences) [42]. Only bacterial ASVs were included in the final output, imported as a Phyloseq object (v 1.26.1) [43] where samples were normalized by rarefying at 50,000 reads. Singletons, doubletons and ASVs with taxonomy assignment confidence < 0.7 were filtered out before the analyses.

#### Alpha and beta diversity

Chao1 and Shannon indexes were computed by the zAMP pipeline. The pairwise dissimilarity was tested with Wilcoxon test or t-test (normal distribution). Beta diversity was assessed based on Bray–Curtis or Jaccard distance and ordinated into non-metric multidimensional scaling (NMDS) plots. The distance between timepoints (longitudinal change) in ventilated and non-ventilated patients was inferred from the Jaccard distance matrix. The dispersion homogeneity and the dissimilarity across subgroups were tested with *betadisper* and *adonis*, respectively.

#### Differential abundance analysis

ASVs prevalent in less than 20 percent of samples were filtered out. We performed linear discriminant analysis effect size (LefSe) for pairwise comparison between COVID-19 and non-COVID-19 pneumonia group with paired samples at day 0 and day 7, by using microbiomeMarker package with Cumulative Sum Scaling (CSS) normalization option. Genera with *p*-value < 0.01 and effect size > 2.5 were selected and visualized. To identify disease severity (ventilation) effect within the longitudinal COVID-19 cohort with varying timepoints and antibiotic treatment conditions, we used negative binomial and zero-inflated mixed model (NBZIMM) [44], which considers the effect of repeated sampling from the same subjects in longitudinal designs. In the mixed model, three fixed effects, “timepoint”, “ventilation” and “antibiotics”, the read counts at genus level and patient identifier were used as inputs. Ventilation-associated genera with *p*-value < 0.01 and effect size > 1 were selected for visualization.

#### Distance-based redundancy analysis

In order to investigate the effect of each variable on the gut microbiota composition, we performed distance-based redundancy analysis (db-RDA) [45], using *capscale* in vegan package. Bray–Curtis distance matrix based on taxa abundance at genus level and metadata were used as inputs in the mixed model. To compare COVID-19 and non-COVID-19 patients, “COVID-19”, “ventilation”, “antibiotics”, “timepoint” and “patient ID” were set as fixed effects. The comparison between ventilated and non-ventilated COVID-19 patients used the same parameters, except “COVID-19”. A triplot was built with the microbiota composition in samples (grey dots), the relative abundance of genera (colored dots) and the effects of selected variables (arrows). Significant variables with *p*-value < 0.05 were highlighted.

### Ethics

This project was conducted in accordance with the Declaration of Helsinki, the principles of Good Clinical Practice and the Swiss Human Research Act (HRA). Informed consent was obtained from all subjects and/or their legal guardian(s). The project received approval from the Ethics Committee of canton Vaud, Switzerland (2020–00755/2017–01820). All data were anonymized before analysis.

## Results

### Demographics

Overall, 57 patients with SARS-CoV-2 infection were enrolled at CHUV during the study period (Figure S1). Median patient age was 68.0 years [IQR 60.0–79.0]. Thirteen (22.8%) were over 80 years old, and 5 (8.8%) were in the 18–49 age group. Median BMI was 25.6 [IQR 23.7–30.6]. The most frequent comorbidities were overweight/obesity and hypertension, found in respectively 31 (54.4%) and 32 (56.2%) of the patients. The median Charlson Comorbidity Index (CCI) was 5.0 [IQR 3.0–6.0]. Twenty-three (40.4%) patients were treated with angiotensin-converting enzyme inhibitors (ACEI) or angiotensin II receptor blockers (ARBs). The characteristics of the 14 patients (24.5%) ventilated were compared to the 43 not ventilated (Table S1). More patients in the ventilated group presented with renal insufficiency (*p* = 0.019). C-reactive protein levels were significantly higher in ventilated compared to non-ventilated patients (medians at respectively 157 and 94, *p* = 0.012). The median duration of symptoms preceding admission (or first positive SARS-CoV-2 test for nosocomial cases) was 6 ± 4 days. The most frequent symptoms at the time of testing were fatigue in 46 (80.7%), cough in 42 (73.7%), dyspnea in 37 (64.9%) and fever in 34 patients (59.6%). The majority of the patients (48, 84.2%) presented a CRB-65 at 0–1. In our population, 8 (14.0%) of all patients received SARS-CoV-2 targeted treatment. The most frequently prescribed medication was remdesivir in 5 patients (8.8%), one of the ventilated patients received tocillizumab. Twenty-three (40.4%) patients received antibiotics. None of the patients was vaccinated for SARS-CoV-2 infection at the time of this study (no vaccine available). At the end of the follow-up, 16 (28%) patients were still hospitalized or transferred to a rehabilitation center, and 35 (61.4%) were discharged home. Six (10.5%) patients died during hospitalization. The only demographic factor significantly associated with mortality was hypertension (51% vs 100% mortality, *p* = 0.03). All the data is presented in supplementary material (Tables S2, S3).

A total of 19 patients were included as control groups; 5 patients intubated without infection nor antibiotic exposure (control group), and 14 patients with pulmonary infection (pneumonia group). Median age was 62.4 years [IQR 33–86] and 11 (57.9%) patients were male. No patient was immunosuppressed, 11 (57.9%) presented comorbid conditions, and no patient was exposed to antibiotics during the last 3 months prior to admission (Table S4). The baseline characteristics of these patients were comparable to the ventilated patients with SARS-CoV-2 regarding age and sex. Moreover, there was no statistical difference regarding the number of enteral and parenteral feeding (*p* = 0.27) (Table S5).

### Comparison of fecal microbiota in COVID-19 and non-COVID-19 patients

To evaluate whether COVID-19 patients presented a signature in their fecal microbiota, we compared their microbiota to non-COVID-19 control patients with ventilatory support in ICU. Nineteen non-COVID-19 ICU patients (control/pneumonia group) were compared with ten COVID-19 ICU patients (COVID-19 group) at day 0 and day 7, the timepoints matched for the comparison.

**Fig. 1:**
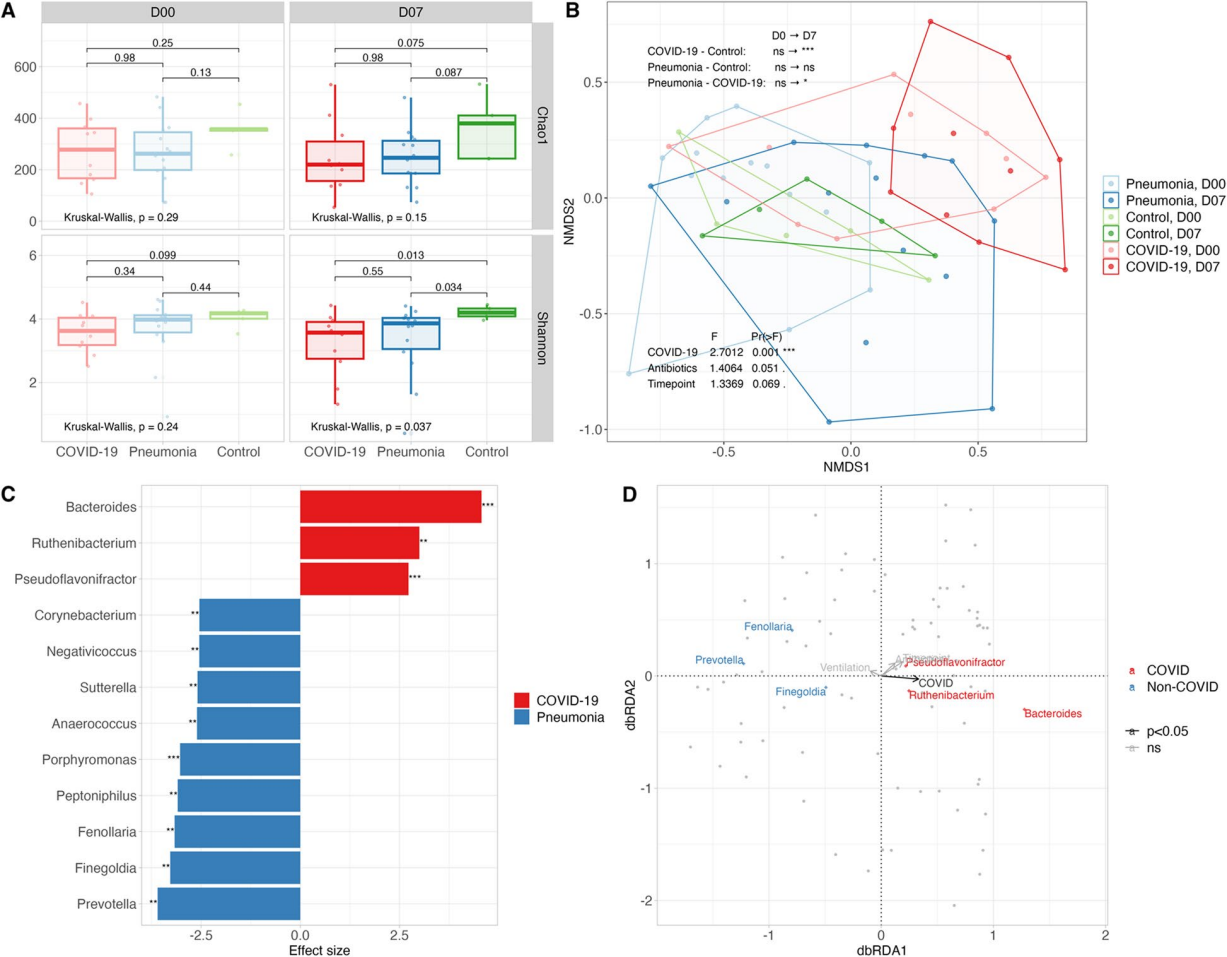
Comparison of the fecal microbiota in COVID-19 and non-COVID-19 patients. **A**, **B** The gut microbiota was compared between ventilated COVID-19 patients with antibiotics (COVID-19 group) to ventilated non-COVID-19 patients who were treated with antibiotics due to ventilation-associated pneumonia (pneumonia group) and those with neither pneumonia nor antibiotics (control group). **A** Chao1 and Shannon alpha diversity indexes in three groups at day 0 (inclusion) and day 7. The results of the Kruskal test and the pairwise Wilcoxon test are indicated within the graphs. **B** Composition of the fecal microbiota in three groups was represented in a non-metric multidimensional scaling (NMDS) plot of Bray–Curtis similarity. The results of pairwise group comparisons at day 0 and day 7 using PERMANOVA, and the effect of three covariates (COVID-19, antibiotics and timepoint) on microbiota composition of the three groups is indicated on the top left and bottom left, respectively. **C**, **D** Differential abundance analysis and distance-based redundancy analysis (db-RDA) in COVID-19 and non-COVID-19 patients. COVID-19 and non-COVID-19 pneumonia patients were compared to evaluate the COVID-19 effect. **C** Differentially abundant taxa identified by linear discriminant analysis effect size (LEfSe) at genus level. The results with *p*-value < 0.01 and effect size (log10) > 2.5 for each group are presented as a bar plot (**p < 0.01, ***p < 0.001). **D** Triplot demonstrating the relationship between the relative abundance of key taxa identified in LEfSe analysis and clinical variables, including COVID-19, ventilation, antibiotics and timepoint. Grey dots represent samples. The length and color of the arrows reflect the variance explained by the clinical variables and the significance of their effect on the gut microbiota composition, respectively. The short distance between the key taxa and the variables inversely indicates strong correlation. The RDA plot was based on Bray–Curtis distance and visualized with type 2 scaling

The richness and evenness of the microbiota were compared among the three groups at day 0 and day 7 separately, using Chao1 and Shannon alpha diversity indexes. At day 0, there was no significant difference among the groups, while at day 7, Shannon diversity appeared significantly lower in the COVID-19 and pneumonia groups than in the control group (COVID-19-control: *p* = 0.013, pneumonia-control: *p* = 0.034, Wilcoxon), as shown in Fig. 1A.

Despite a significant difference in variance between COVID-19 and control groups (COVID-19-control: 0.036, COVID-19-pneumonia: ns, pneumonia-control: ns, betadisper) in Bray–Curtis distance (Fig. 1B), the dissimilarity between the groups was non-significant at day 0 in all pairwise comparisons. At day 7, however, the COVID-19 group became significantly distinct from the pneumonia group (*p* = 0.028, PERMANOVA; ns, betadisper) and the control group (*p* = 0.003, PERMANOVA; *p* = 0.04, betadisper), while the pneumonia group remained largely overlapped with the control group with heterogeneous dispersion (ns, PERMANOVA; *p* = 0.0127, betadisper). Among the different factors potentially influencing the microbiota, only COVID-19 had a significant effect (F = 2.7012, *p* = 0.001, PERMANOVA), but not antibiotics (F = 1.4064, *p* = 0.051, PERMANOVA) or timepoint (F = 1.3369, *p* = 0.069).

The effect of COVID-19 on bacterial composition in the feces was assessed by identifying differentially abundant genera in the COVID-19 group compared to the pneumonia group. The COVID-19 group was characterized by an increased abundance of *Bacteroides, Ruthenibacterium* and *Pseudoflavonifractor*, while pneumonia patients harbored a higher abundance of *Prevotella, Finegoldia, Fenollaria, Peptoniphilus, and Porphyromonas* (Fig. 1C). At phylum level, the *Bacteroidetes*/*Firmicutes* ratio was significantly increased (*p* = 3.8e-05, Wilcoxon).

In order to disentangle the mixed effects of multiple factors affecting the microbiota, a complex model was built using distance-based redundancy analysis (db-RDA) with different variables (COVID-19, ventilation, antibiotics, timepoint and patient), including all samples from both ventilated and non-ventilated patients at day 0 and day 7 (Fig. 1D). As expected, the patient significantly (F = 1.7333, *p* = 0.001) impacted the microbiota, in line with the concept of individual microbiota fingerprint. Interestingly, among the variables of interest, only COVID-19 showed a significant effect (F = 3.7298, *p* = 0.005), but ventilation, antibiotics and timepoint appeared as non-significant variables. Furthermore, the correlation between COVID-19 and taxa abundance was consistent with the results of differential abundance analysis (Fig. 1C).

### COVID-19 severity and fecal microbiota

**Fig. 2:**
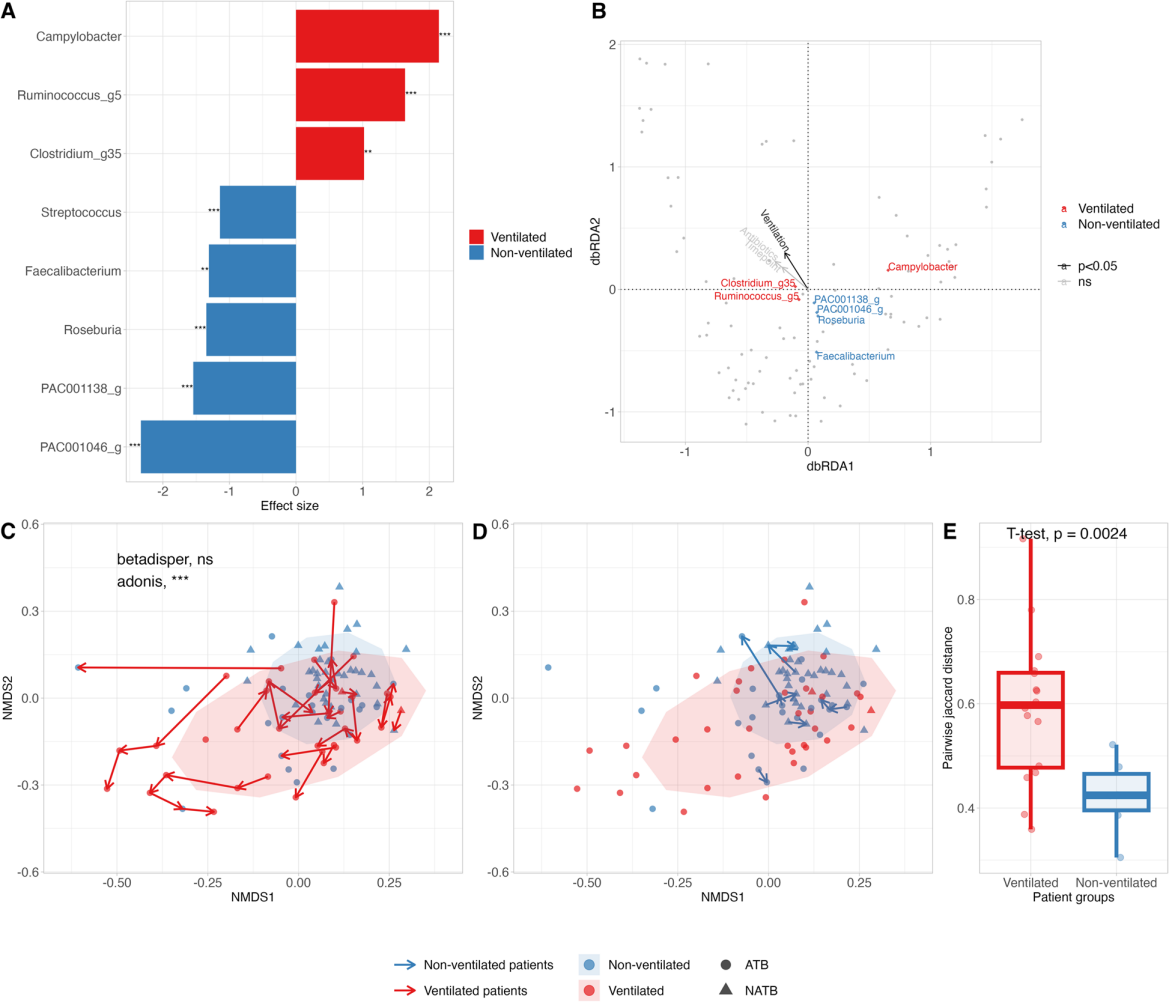
Impact of COVID-19 severity on the taxa composition and longitudinal changes of the gut microbiota. **A**, **B** Differential abundance analysis and db-RDA analysis in ventilated and non-ventilated COVID-19 patients. **A** Genera differentially abundant among the two groups were identified by a complex model including ventilation, antibiotics and timepoint with longitudinal sampling correction using negative binomial and zero-inflated mixed model (NBZIMM). The taxa with *p*-value < 0.01 and effect size (log10) > 2.5 for each group are presented in the bar plot (**p < 0.01, ***p < 0.001). **B** RDA triplot based on Bray–Curtis distance visualized with type 2 scaling. The correlation between relative abundance of key taxa identified by NBZIMM and clinical variables—ventilation, antibiotics and timepoint—is represented. **C**, **D** Beta diversity of ventilated and non-ventilated groups was represented using NMDS plot based on Jaccard distance. Beta diversity at all time points is displayed with ellipses, where the color of each dot indicates the location of the patient at the sampling time point. Samples from patients in antibiotics (ATB) and non-antibiotics (NATB) groups are represented as circles and triangles, respectively. Subsequent samples from the same patients were linked by arrows. Ventilated patients (red arrow) have been ventilated at more than one time point and non-ventilated patients (blue arrow) had no ventilation history during their hospitalization. The four samples of one patient, who had been continuous antibiotic treatment with multiple antibiotics for 2 months before inclusion, clustered distantly from other samples and were hence excluded from the representation. **E** The mean Jaccard distance, a binary measure of bacterial presence and absence, between time-ordered samples for each patient was compared between ventilated and non-ventilated groups

To investigate the difference in microbial composition between ventilated and non-ventilated patients with varying number of samples and timepoints, differentially abundant bacterial genera were identified with longitudinal repeated sampling correction (Fig. 2A). In ventilated patients, we observed an increase in the abundance of *Campylobacter*, *Ruminococcus* and *Clostridium* and a decrease in the abundance of two unassigned genera (PAC001138 and PAC001046) in *Lachnospiraceae* family, *Roseburia*, *Faecalibaterium* and *Streptococcus*. The effects of different variables (ventilation, antibiotics, timepoint and patient) were assessed with db-RDA in COVID-19 patients (Fig. 2B). Apart from the patient variable (F = 1.4062, *p* = 0.001, ANOVA), only ventilation showed a significant effect (F = 2.6901, *p* = 0.006, ANOVA) on microbial composition, whereas no significance was observed for antibiotics and timepoint.

Changes in fecal microbiota diversity were compared between COVID-19 patients with and without ventilation to assess the effect of disease severity. A longitudinal decrease in alpha diversity was observed, particularly in ventilated patients, from day 0 to day 14 (Figure S2) albeit not statistically significant due the large variations within group and timepoints.

Longitudinal changes in the fecal microbiota composition across samples from ventilated and non-ventilated patients were then assessed using Jaccard distance matrix. At inclusion, both ventilated and non-ventilated patients showed a similar microbiota composition (ns, PERMANOVA) and variance (ns, betadisper) (Figure S3). However, ventilated patients presented rapid alterations of the microbiota (*p* < 0.001, PERMANOVA; ns, betadisper) (Fig. 2C-D). The mean distance between subsequent samples from the same patient was significantly higher in the ventilated group, implying greater alterations over time in the gut microbiota of ventilated patients than non-ventilated patients (Fig. 2E).

### Association of SARS-CoV-2 excretion with severity and microbiota composition

**Fig.3:**
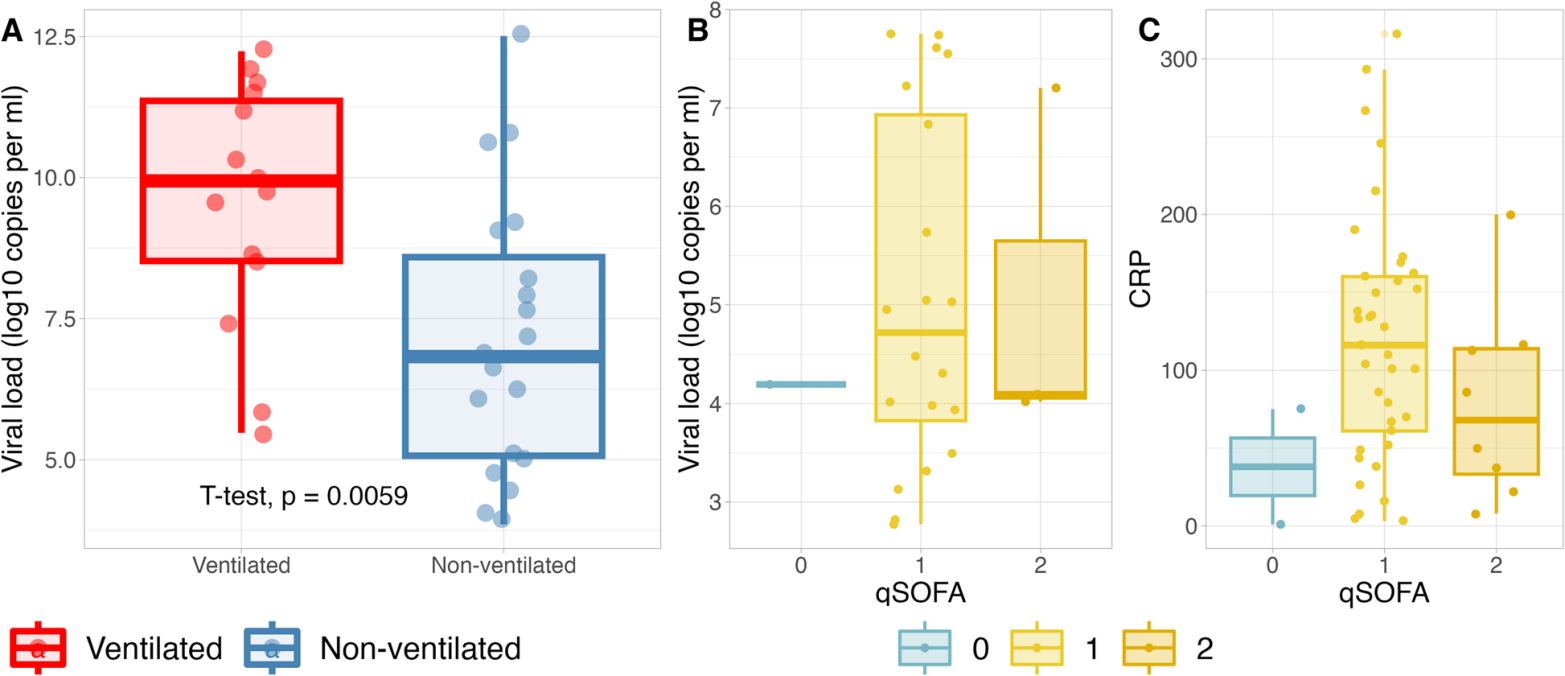
Viral load of PCR positive samples in association with ventilation and COVID severity. **A** Viral load is higher in ventilated patients than non-ventilated patients (*p* = 0.0059, T-test). **B** Viral load according to qSOFA score. **C** CRP values according to qSOFA score

**Fig. 4:**
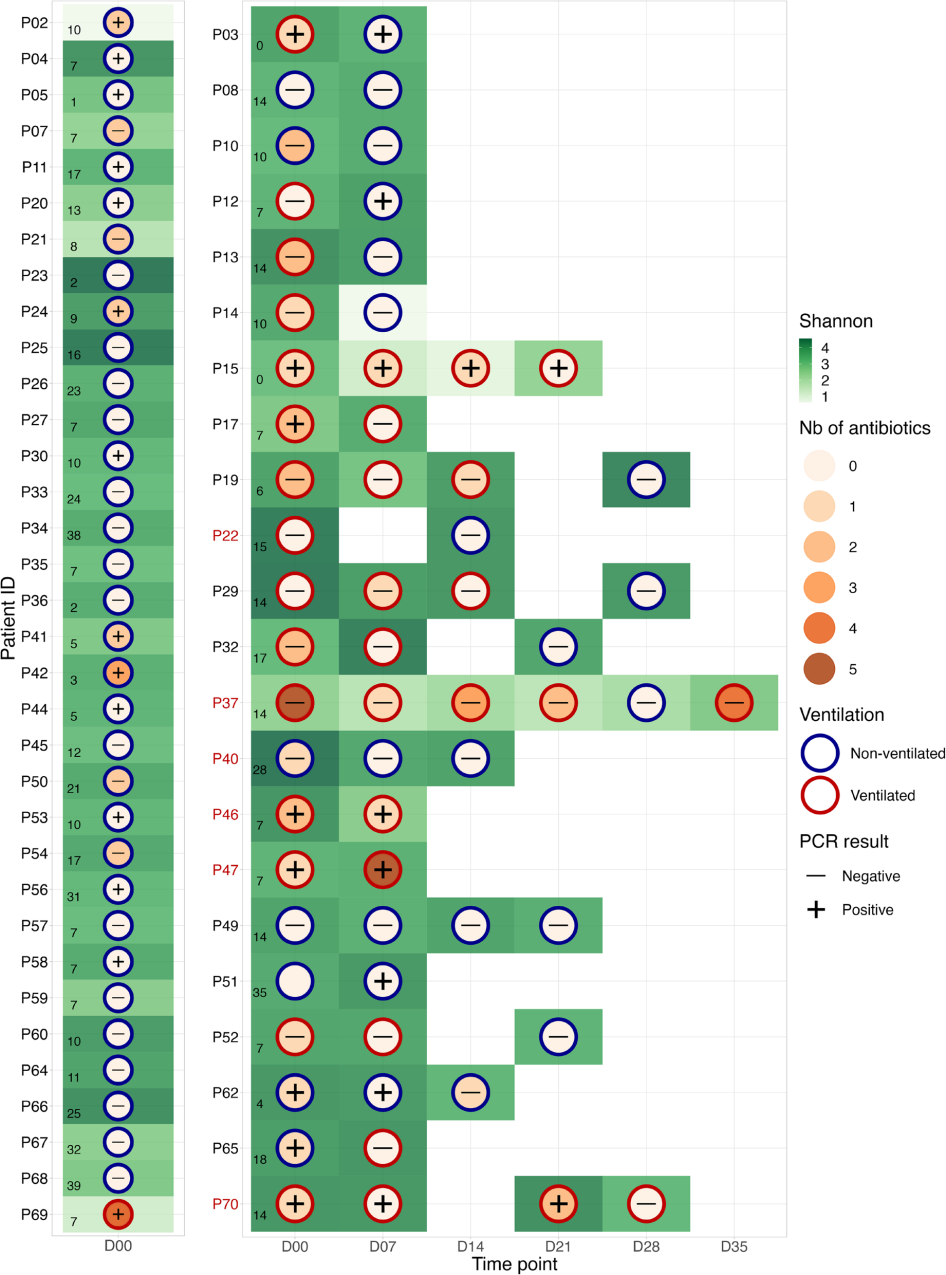
Timeline of sampling with various information. Patients only day 0 samples are gathered on the left panel while those with multiple samples are shown on the right panel. Patients who died are labelled in red. The border of circles represents ventilated (red) and non-ventilated (blue) state, where SARS-CoV-2 PCR results in rectal swabs are written as positive ( +) and negative (-). The number of days after symptom onset is indicated at the left bottom side of day 0. One sample from P51 is lacking the PCR result. The gradient color inside (orange scale) and outside (green scale) circles show the number of treated antibiotics and Shannon index, respectively

To evaluate the excretion of SARS-CoV-2 particles in patients’ gastrointestinal tract and its association with disease severity, a SARS-CoV-2 qPCR was performed in rectal swabs. The proportion of qPCR-positive patients in ventilated and non-ventilated patients was comparable (38.9% and 36.5%, respectively). The cycle threshold (CT) value of positive samples was lower and hence the viral load was significantly higher (*p* = 0.0011, t-test) in ventilated patients compared to non-ventilated patients (Fig. 3A-B). Interestingly we observed a dissociation between viral load, severity assessed by the qSOFA and inflammation measured by C-reactive protein (CRP) (Fig. 3C-D). The summary of samples and patient conditions, such as alpha diversity, ventilation, antibiotics and PCR results, is visualized in Fig. 4.

## Discussion

The clinical characteristics of our cohort, recruited before vaccination and SARS-CoV-2 Omicron variant, are comparable to other studies investigating the gut microbiota in COVID-19 patients [46]. The most common symptoms observed in our cohort such as fatigue, cough, dyspnea and fever are also frequently reported in the literature [46–48]. Based on our longitudinal and cross-sectional cohort, we were able to evaluate and compare the changes in the gut microbiota in relation to COVID-19 and its severity.

Comparing the fecal microbiota between COVID-19 and non-COVID-19 patients, we found that alpha diversity decreased over time in both non-COVID-19 pneumonia and COVID-19 groups compared to control group. However, the temporal changes in the microbiota of COVID-19 patients, in contrast to the pneumonia patients, made their composition significantly distinct from that of control group. In the comparative analysis of differentially abundant taxa in COVID-19 and pneumonia groups under the same conditions of ventilation and antibiotic treatment, COVID-19 fecal microbiota was characterized by *Bacteroides*, while non-COVID-19 by *Prevotella*. As prevalent bacterial genera in the gut, *Bacteroides* and *Prevotella* have been studied as enterotypes with antagonistic metabolic functions [49]. In particular, Mejía-León et al. have focused on comparing their roles in the immune system, suggesting that *Bacteroides* is associated with leaky gut (increased intestinal permeability), pro-inflammatory responses, whereas *Prevotella* is associated with butyrate production, which is known to induce mucin synthesis for tight junctions in the intestinal epithelium, and anti-inflammatory responses [50]. We also observed that the *Bacteroidetes/Firmicutes* ratio was significantly higher in COVID-19 than in pneumonia patients. An increase in this ratio in the gut is known to be associated with inflammatory bowel disease (IBD) [51]. Khan et al. [52] also found an increase in the ratio in COVID-19 patients and suggested that the depletion of the fiber-utilizing bacteria such as *Faecalibacterium* and *Prevotella*, and an increase in *Bacteroidetes* led to the increase in the ratio, suggesting an association between gut microbiota dysbiosis and COVID-19 disease severity.

This observation was also consistent when comparing COVID-19 severity among COVID-19 patients, where butyrate-producing bacteria (*Lachnospiraceae* family, *Roseburia* and *Faecalibaterium)* were largely reduced in ventilated COVID-19 patients than in non-ventilated COVID-19 patients. We also observed, in COVID-19 patients, a decreased alpha diversity in ventilated and non-ventilated groups over time with a more drastic decrease in ventilated patients. Taken together with the diversity analysis results above, the temporal changes in fecal microbiota seem greater in COVID-19 and severe COVID-19 patients, compared to non-COVID-19 and moderate COVID-19 patients, respectively. This suggests that COVID-19 and its severity are positively associated with gut microbiota instability, in line with a study of Schult et al. [53].

Several studies already focused on the gut microbiota in COVID-19 patients (for a review, see Liu et al. [5]) and our results are partially concordant with the literature such as in a cohort of 69 COVID-19 critically ill patients showing a reduced alpha diversity [22]. An observational cohort study comprising 62 COVID-19 patients, 40 healthy controls and 33 seasonal flu also reported a decrease of alpha diversity in the gut microbiota and clear differences in beta diversity compared to control groups [19]. In this study the authors used healthy controls as a reference and showed that the abundance of members of the genera *Streptococcus, Clostridium, Lactobacillus*, and *Bifidobacterium* was increased, whereas the abundance of *Bacteroidetes, Roseburia, Faecalibacterium, Coprococcus*, and *Parabacteroides* was decreased in COVID-19 patients. Another small cohort including 23 patients showed that gut microbial richness was reduced in COVID-19 for ICU patients compared to patients in the general wards [54]. At the family level, COVID-19 patients had an enrichment in *Enterococcaceae, Coriobacteriaceae, Lactobacillaceae, Veillonellaceae, Porphyromonadaceae* and *Staphylococcaceae*, contrasting with a decrease in *Bacteroidaceae, Lachnospiraceae* and *Ruminococcaceae, Prevotellaceae and Clostridiaceae*. These patients were comparable to our population with a median age of 73 years-old and a median CCI at 5 compared to respectively 68 years-old and 5 in our study. Finally, a two-hospital cohort study analyzed stool samples from 87 COVID-19 patients and 78 controls, as well as serial stool samples from 27 patients up to 30 days after clearance of the virus [6]. Similar to our findings, the authors did not show any overall difference in diversity between COVID-19 and non-COVID-19 patients at the first sampling timepoint. They also reported that members of the *Bacteroidetes* were more abundant and *Actinobacteria* depleted in patients with COVID-19 compared to non-COVID-19 individuals. Compositional difference testing revealed an underrepresentation of gut commensals with immunomodulatory properties such as *Faecalibacterium prausnitzii* and *Eubacterium rectale* [6]*,* but also *Lachnospiraceae* [23], generally driven by antibiotics administration, in COVID-19 patients. Consistent with the study of Zuo et al. [23], we observed a separation of patients exposed to antibiotics (COVID-19 and non-COVID-19) compared to patients without antibiotics.

In our study, the excretion of the virus in the stool measured as the percentage of positive stools was comparable in ventilated and non-ventilated patients (38.9% and 36.5%, respectively). In a cohort of 59 patients in Hong-Kong, 15/59 (25.4%) patients presented GI symptoms and 9 (15.3%) had stool that tested positive for SARS-CoV-2 RNA [8]. A meta-analysis of 60 studies with 4243 patients observed gastro-intestinal symptoms in respectively 11.8% and 17.1% of non-severe and severe patients, while viral RNA was detected in stool samples from 48.1% of the patients [8]. Pan et al. found comparable results with positive stool samples in 53% of the cases [55]. In the Hong Kong study [8], median fecal viral load was 5.1 log_10_ cp/ml in patients with diarrhea, which is lower than the 7.9 log_10_ cp/ml (7 patients) observed in our cohort. In another cohort, Zuo et al. also showed a correlation between dysbiosis and infectivity of COVID-19; stool samples with high infectivity presented a significant increase of *Collinsella aerofaciens, Collinsella tanakaei, Streptococcus infantis* and *Morganella morganii* compared to samples with low COVID-19 infectivity [56].

In the last part of our study, we analyzed disease severity assessed with the qSOFA score. First of all, we did not observe any significant difference in qSOFA between ventilated and non-ventilated patients, nor in the viral load, confirming that this classical tool to assess severity is questionable for ICU patients. Indeed, another study previously showed that qSOFA failed to identify patients with a poor outcome in 52 critically ill ICU patients [57]. We also observed a slight correlation of viral load and CRP with the qSOFA score; although not significant, viral load and CRP increased when qSOFA increased. In a multicenter prospective cross-sectional study with 115 COVID-19 patients [10], severity was negatively associated in the multivariate analysis with Shannon diversity index (OR = 2.85) and C-reactive protein level higher than 96.8 mg/L (OR = 3.45). Consistent with another Swiss cohort of 145 COVID-19 patients [48] with similar characteristics to our cohort, CRP was significantly higher in ventilated patients.

While our study showed overall concordant results with existing literature, the limited number of samples and patients in each group limited the statistical power of some comparisons and the strength of the conclusions that can be drawn. Indeed, even though the patients were prospectively enrolled, the first wave was associated with many logistics issues for basic research, preventing larger patient inclusions. Finally, our analysis only reflects the composition of the microbiota and does not evaluate the function and the immune response, which are also a key factor in the gut-lung axis [16].

## Conclusions

We observed associations between the gut microbiota and SARS-CoV-2 infection, severity, and antibiotics treatment. The composition of the gut microbiota in patients with COVID-19 became significantly distinct from the control group over time whereas the pneumonia group retained a similar composition. COVID-19 patients were mainly characterized by increased *Bacteroides* and decreased *Prevotella* and disease severity was negatively correlated with butyrate-producing bacteria. COVID-19-induced profile may represent a signature of the disease that should be further studied to better understand the physiopathological processes influencing and triggered by the gut microbiota, notably on the response to infection, and propose targeted therapeutic responses.

### Supplementary Information

**Additional file 1: Figure S1.** Flow chart of the study. **Figure S2.** Alpha diversity changes over time in ventilated and non-ventilated groups. **Figure S3.** Jaccard beta-diversity of ventilated and non-ventilated patients at day 0 depicted on NMDS.

**Additional file 2: Table S1.** Demographic characteristics of participants based on the ventilatory status in the COVID-19 cohort. **Table S2.** COVID-19 symptoms, status and treatment of participants based on the ventilatory status in the COVID-19 cohort. **Table S3.** Risk factors of COVID-19 infected participants. **Table S4.** Demographic characteristics of Non-COVID-19 ventilated patients. **Table S5.** Nutrition characteristics of the patients.

## Data Availability

The datasets used and/or analyzed during the current study are available from the European Nucleotide Archive (ENA): PRJEB61723/ERP146805 and PRJEB61722/ERP146804.

## Abbreviations

SARS-CoV-2: Severe acute respiratory syndrome coronavirus 2
COVID-19: Coronavirus disease
ACE2: Angiotensin-converting enzyme 2
ICU: Intensive care unit
CHUV: Lausanne University Hospital
RT-PCR: Reverse-transcriptase-polymerase-chain-reaction
VAP: Ventilator-associated pneumonia
EHR: Electronic health record
CCI: Charlson Comorbidities Index
SOFA score: Sequential Organ Failure Assessment score
qSOFA score: Quick SOFA score
CRB-65 score: Confusion—Respiratory rate—Blood pressure—age ≥ 65 years score
NEWS score: National early warning score
CRP: C-Reactive Protein
PCT: Procalcitonin
AKI: Acute Kidney Injury
ARDS: Acute Respiratory Distress Syndrome
EHR: Electronic Health Records
eCRF: Electronic clinical report form
SD: Standard deviation
MV: Mechanical ventilation
IQR: Interquartile range
CT: Cycle threshold
ASVs: Amplicon sequence variants
LefSe: Linear discriminant analysis effect size
CSS: Cumulative sum scaling
NBZIMM: Negative binomial and zero-inflated mixed model
db-RDA: Distance-based redundancy analysis
HRA: Human Research Act
ACEI: Angiotensin-converting enzyme inhibitors
ARBs: Angiotensin II receptor blockers
IBD: Inflammatory bowel disease
