# Supplementary material for: Temporal changes in fecal microbiota of patients infected with COVID-19: a longitudinal cohort
Source: BMC Infect Dis. 2023 Aug 18;23:537. doi: 10.1186/s12879-023-08511-6 (PMC10436399; doi:10.1186/s12879-023-08511-6)
Supplement: Supplementary file 1 — Additional file 1: Figure S1. Flow chart of the study. Figure S2. Alpha diversity changes over time in ventilated and non-ventilated groups. Figure S3. Jaccard beta-diversity of ventilated and non-ventilated patients at day 0 depicted on NMDS. [file 12879_2023_8511_MOESM1_ESM.pdf]

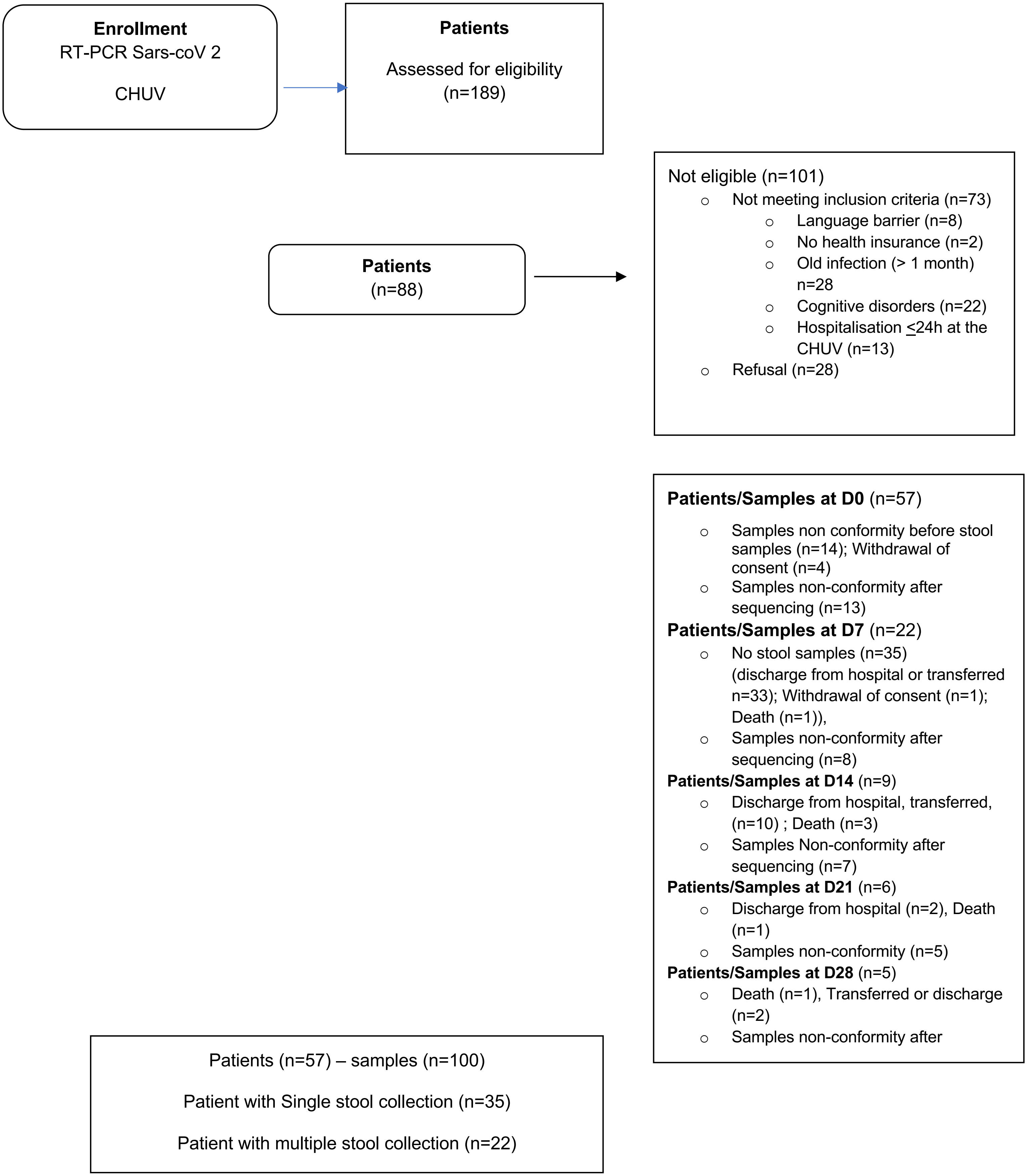

Figure S1: Flow chart of the study

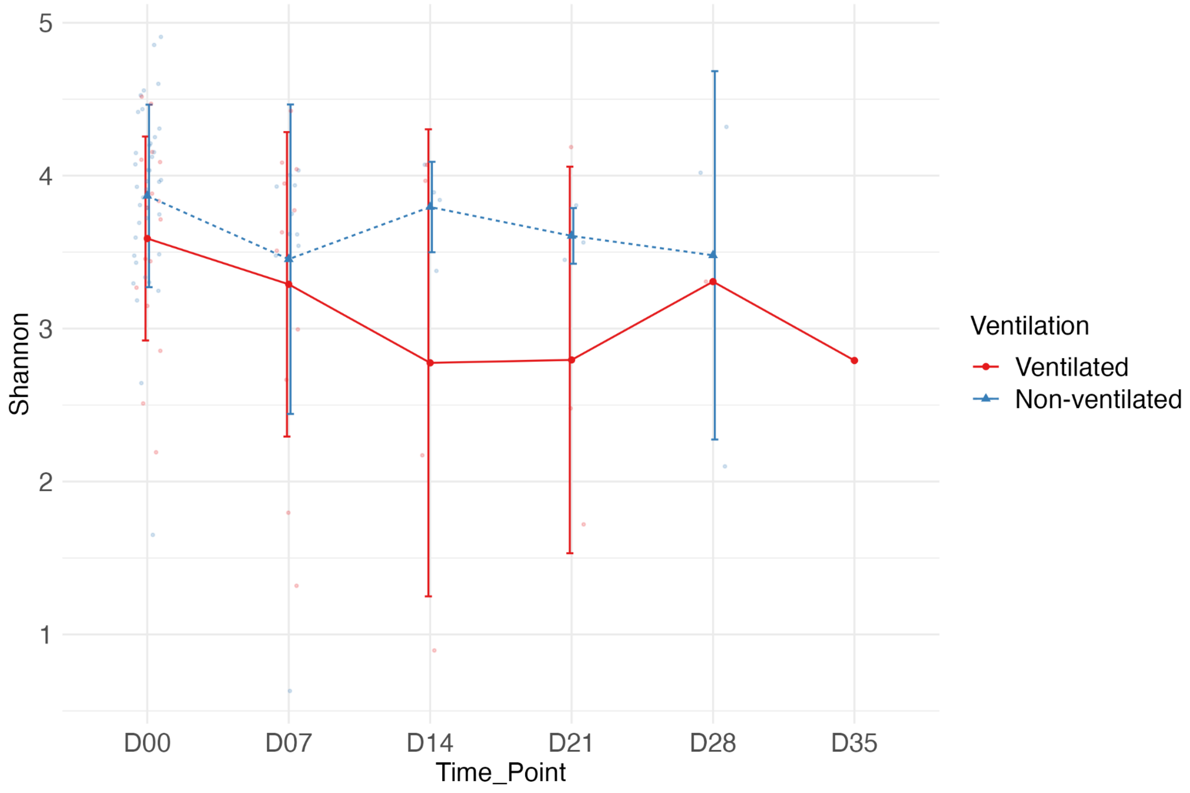

Figure S2. Alpha diversity changes over time in ventilated and non-ventilated groups. Shannon index reflects the richness and the evenness of the bacterial community. The differences between two groups at each point and between timepoints in each group were statistically non-significant.

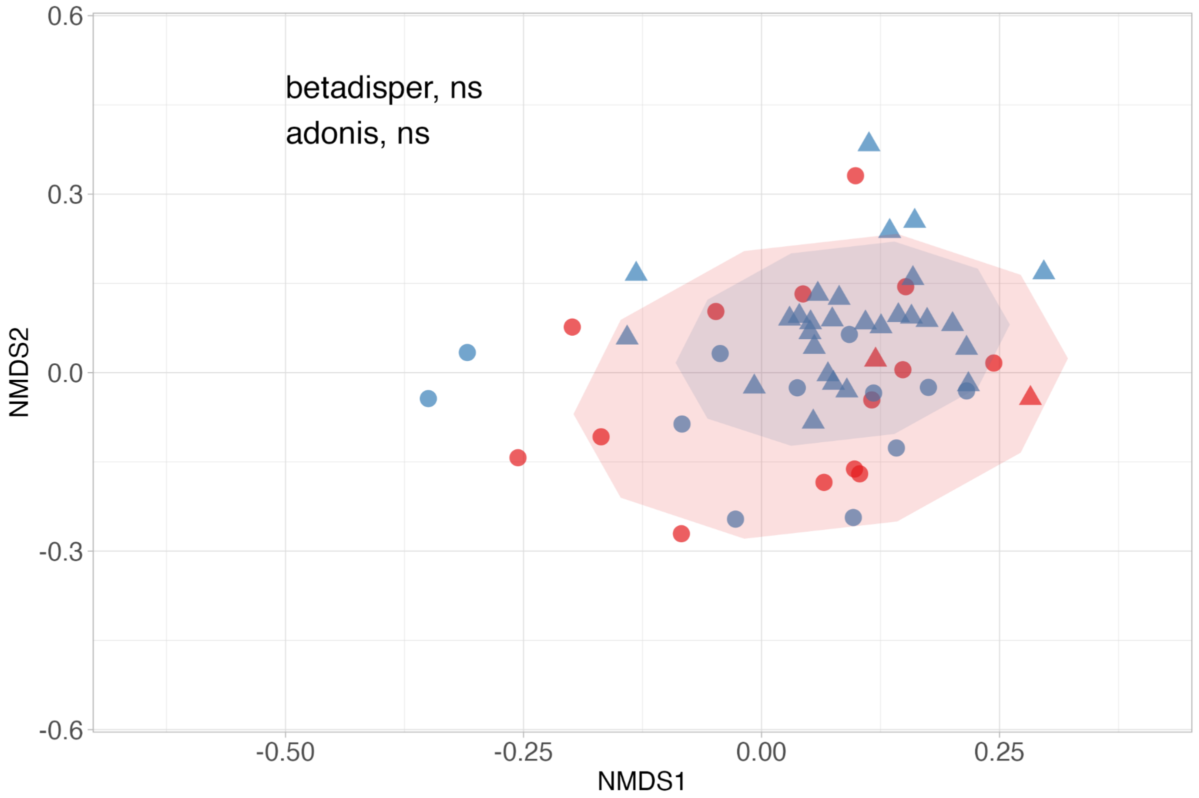

Figure S3. Jaccard beta-diversity of ventilated and non-ventilated patients at day 0 depicted on NMDS. The groups of ventilated and non-ventilated patients show a similar variance but a significant difference in composition.
