# Supplementary material for: Temporal changes in fecal microbiota of patients infected with COVID-19: a longitudinal cohort
Source: BMC Infect Dis. 2023 Aug 18;23:537. doi: 10.1186/s12879-023-08511-6 (PMC10436399; doi:10.1186/s12879-023-08511-6)
Supplement: Supplementary file 2 — Additional file 2: Table S1. Demographic characteristics of participants based on the ventilatory status in the COVID-19 cohort. Table S2. COVID-19 symptoms, status and treatment of participants based on the ventilatory status in the COVID-19 cohort. Table S3. Risk factors of COVID-19 infected participants. Table S4. Demographic characteristics of Non-COVID-19 ventilated patients. Table S5. Nutrition characteristics of the patients. [file 12879_2023_8511_MOESM2_ESM.pdf]

Table S1: Demographic characteristics of participants based on the ventilatory status in the COVID-19 cohort

	**Not ventilated**[1] **n= 43**	**Ventilated**[1] **n=14**	**Overall**[1] **n=57**	**p-value**[2]
**Demographic**				
Age, years	72 (62, 80)	63 (58, 73)	68 (60, 79)	0.069
Sex				0.5
Female	32.6% (14)	21.4% (3)	29.8% (17)	
Male	67.4% (29)	78.6% (11)	70.2% (40)	
Body mass index (BMI)	25.5 (23.3, 28.2)	29.2 (25.3, 36.0)	25.6 (23.7, 30.6)	0.053
Smoking habits	25.6% (11)	35.7% (5)	28.1% (16)	0.5
ACE inhibitors	37.2% (16)	50% (7)	40.4% (23)	0.4
Charlson comorbidity index	5.0 (3.0, 6.0)	4.5 (2.25, 6.75)	5.0 (3.0, 6.0)	>0.9
Overweight and obese (BMI>25)	53.5% (23)	57.1% (8)	54.4% (31)	0.8
Hypertension	53.5% (23)	64.3% (9)	56.1% (32)	0.5
Use of vasopressors	0% (0)	21% (3)	5% (3)	>0.9
**Laboratory data**				
White blood cell count, $x10^9$ per L	6.0 (4.3, 8.1)	7.1 (2.8, 8.3)	6.3 (3.9, 8.1)	>0.9
Platelets	238 (182, 314)	222 (198, 282)	234 (183, 313)	>0.9
D-dimers	908 (669, 1181)	1009 (580, 1058)	936 (580, 1163)	0.9
Creatinin	91 (78, 139)	95 (67, 134)	92 (77, 138)	0.9
C-Reactive protein	94 (40, 137)	157 (110, 267)	104 (50, 154)	0.012
AKIN on admission				0.019
No	67.4% (29)	35.7% (5)	59.6% (34)	
AKIN I	20.9% (9)	14.3% (2)	19.3% (11)	
AKIN II	4.7% (2)	14.3% (2)	7% (4)	
Missing	7% (3)	35.7% (5)	14% (8)	

[1]Median (IQR) ; % (n)
[2]Wilcoxon rank sum test; Fisher's exact test; Pearson's Chi-squared test

Table S2: COVID-19 symptoms, status and treatment of participants based on the ventilatory status in the COVID-19 cohort

	Not ventilated[1] n= 43	Ventilated[1] n=14	Overall[1] n=57	p-value[2]
**Symptoms**				
Temperature	38.1 (37.1, 38.5)	38.2 (37.6, 38.8)	38.1 (37.2, 38.8)	0.5
Cough	72.1% (31)	78.6% (11)	73.7% (42)	0.7
Fatigue	79.1% (34)	85.7% (12)	80.7% (46)	0.7
Anosmia/Dysgueusia	16.3% (7)	7.1% (1)	14% (8)	0.7
Dyspnea	58.1% (25)	85.7% (12)	64.9%(37)	0.11
Diarrhea	30.2% (13)	21.4% (3)	28.1% (16)	0.7
**Status**				
Heart rate	93 (80, 98)	96 (84, 102)	94 (81, 99)	0.4
Systolic blood pressure	115 (104, 132)	127 (119, 133)	120 (106, 132)	0.14
SpO_2 on admission	97 (95, 98)	94 (93, 95)	97 (95, 98)	0.051
CRB 65				0.6
0-1	83.7% (36)	85.7% (12)	84.2% (48)	
2	16.3% (7)	14.3% (2)	15.8% (9)	
3-5	0	0	0	
Type of discharge				0.02
Alive	97.7% (42)	64.3% (9)	89.5% (51)	
Dead	2.3% (1)	35.7% (5)	10.5% (6)	
**Treatment**				
Remdesivir	2.3% (1)	28.6% (4)	8.8% (5)	0.011
Other COVID-directed therapy[3]	4.6% (2)	7.1% (1)	5.2% (3)	0.2
Any antibiotic[4]	25.6% (11)	85.7% (12)	40.4% (23)	<0.001

[1]Median (IQR) ; % (n)
[2]Wilcoxon rank sum test; Fisher's exact test; Pearson's Chi-squared test
[3]Molecule among hydroxychloroquine, lopinavir, tocillizumab
[4]Molecule among co-amoxicilline, piperacillin-tazobactam, ceftriaxone, cefepime, meropenem, levofloxacin

Table S3: Risk factors of COVID-19 infected participants

**Characteristics**	**Alive**[1] **n= 51**	**Dead**[1] **n=6**	**Overall**[1] **n=57**	**p-value**[2]
Age	68 (60, 79)	74 (67, 76)	68 (60, 79)	0.4
Sex				0.5
Female	33.3% (17)	0% (0)	29.8% (17)	
Male	66.7% (34)	100% (6)	70.2% (40)	
Body mass index	25.8 (23.8, 30.6)	24.4 (22.6, 26.1)	25.6 (23.7, 30.6)	0.3
Smoking habits	27.4% (14)	33.3% (2)	28.1% (16)	0.7
ACE inhibitors	43.1% (22)	16.7% (1)	40.4% (23)	0.4
Charlson comorbidity index	4.0 (2.5, 6.0)	5.5 (5.0, 6.75)	5.0 (3.0, 6.0)	>0.9
Overweight and obese	58.8% (30)	16.7% (1)	54.4% (31)	0.8
Hypertension	51.0% (26)	100% (6)	56.1% (32)	0.03

[1]Median (IQR) ; % (n)
[2]Wilcoxon rank sum test; Fisher's exact test; Pearson's Chi-squared test

Table S4: Demographic characteristics of Non-COVID-19 ventilated patients

	**Control**[1] **n=5**	**Pulmonary**[1] **n=14**	**Overall**[1] **n=19**
Age	77 (33, 86)	63 (36, 86)	64 (33, 86)
Sex			
Female	60.0% (3)	35.7% (5)	42.1% (8)
Male	40.0% (2)	64.3% (9)	57.9% (11)
Body mass index	27.7 (24.0, 31.0)	26.1 (24.3, 26.5)	26.1 (24.1, 27.3)
Temperature	37.4 (35.5, 38.4)	36.2 (34.1, 38.3)	36.7 (34.1, 38.4)
Any comorbidities	60.0% (3)	57.1% (8)	57.9% (11)
Smoking habits	0% (0)	21.4% (3)	15.8% (3)
Leucocytes	14.8 (6.60, 22.0)	14.1 (8.40, 22.1)	14.6 (6.60, 22.1)
Creatinin	115 (65.0, 316)	111 (42.0, 472)	111 (42.0, 472)
Use of vasopressors	80% (4)	71% (10)	73% (14)

[1]Median (IQR) ; % (n)

Table S5: Nutrition characteristics of the patients

	COVID-19 patients (n=57)		Non-COVID-19 patients (n=19)
	Not ventilated	Ventilated	
**Oral feeding**	97.7% (42)	7.1% (1)	-
**Parenteral feeding**	0	35.7% (5)	63.2% (12)
**Enteral feeding**	2.3% (1)	71.4% (10)	89.4% (17)
